# Integrating phenotypic and expression profiles to map arsenic-response networks

**DOI:** 10.1186/gb-2004-5-12-r95

**Published:** 2004-11-29

**Authors:** Astrid C Haugen, Ryan Kelley, Jennifer B Collins, Charles J Tucker, Changchun Deng, Cynthia A Afshari, J Martin Brown, Trey Ideker, Bennett Van Houten

**Affiliations:** 1 Laboratory of Molecular Genetics, National Institute of Environmental Health Sciences, NIH, Research Triangle Park, NC 27709, USA; 2 Department of Bioengineering, University of California San Diego, 9500 Gilman Drive, La Jolla, CA 92093-0412, USA; 3 National Center for Toxicogenomics, Microarray Center, National Institute of Environmental Health Sciences, NIH, Research Triangle Park, NC 27709, USA; 4 Department of Radiation Oncology, Stanford University School of Medicine, 269 Campus Drive West, Stanford, CA 94305, USA

## Abstract

**Background:**

Arsenic is a nonmutagenic carcinogen affecting millions of people. The cellular impact of this metalloid in *Saccharomyces cerevisiae *was determined by profiling global gene expression and sensitivity phenotypes. These data were then mapped to a metabolic network composed of all known biochemical reactions in yeast, as well as the yeast network of 20,985 protein-protein/protein-DNA interactions.

**Results:**

While the expression data unveiled no significant nodes in the metabolic network, the regulatory network revealed several important nodes as centers of arsenic-induced activity. The highest-scoring proteins included Fhl1, Msn2, Msn4, Yap1, Cad1 (Yap2), Pre1, Hsf1 and Met31. Contrary to the gene-expression analyses, the phenotypic-profiling data mapped to the metabolic network. The two significant metabolic networks unveiled were shikimate, and serine, threonine and glutamate biosynthesis. We also carried out transcriptional profiling of specific deletion strains, confirming that the transcription factors Yap1, Arr1 (Yap8), and Rpn4 strongly mediate the cell's adaptation to arsenic-induced stress but that Cad1 has negligible impact.

**Conclusions:**

By integrating phenotypic and transcriptional profiling and mapping the data onto the metabolic and regulatory networks, we have shown that arsenic is likely to channel sulfur into glutathione for detoxification, leads to indirect oxidative stress by depleting glutathione pools, and alters protein turnover via arsenation of sulfhydryl groups on proteins. Furthermore, we show that phenotypically sensitive pathways are upstream of differentially expressed ones, indicating that transcriptional and phenotypic profiling implicate distinct, but related, pathways.

## Abstract 2

By integrating phenotypic and transcriptional profiling and mapping the data onto metabolic and regulatory networks, it was shown that arsenic probably channels sulfur into glutathione for detoxification, leads to indirect oxidative stress by depleting glutathione pools, and alters protein turnover via arsenation of sulfhydryl groups on proteins.

## Background

Global technologies in the budding yeast *Saccharomyces cerevisiae *have changed the face of biological study from the investigation of individual genes and proteins to a systems-biology approach involving integration of global gene expression with protein-protein and protein-DNA information [1]. These data, when combined with phenotypic profiling of the deletion mutant library of nonessential genes, allow an unparalleled assessment of the responses of yeast to environmental stressors [2-4]. In this study, we used these two genomic approaches to study the response of yeast to arsenic, a toxicant present worldwide, affecting millions of people [5].

Arsenic, a ubiquitous environmental pollutant found in drinking water, is a metalloid and human carcinogen affecting the skin and other internal organs [6]. It is also implicated in vascular disorders, neuropathy, diabetes and as a teratogen [7]. Furthermore, arsenic compounds are also used in the treatment of acute promyelocytic leukemia [8-10]. Consequently, the potential for future secondary tumors resulting from such therapy necessitates an understanding of the mechanisms of arsenic-mediated toxicity and carcinogenicity. However, even though a number of arsenic-related genes and processes related to defective DNA repair, increased cell proliferation and oxidative stress have been described, the exact mechanisms of arsenic-related disease remain elusive [11-19]. This is, in part, due to the lack of an acceptable animal model that faithfully recapitulates human disease [15].

A number of proteins involved in metalloid detoxification have been described in different organisms, including *Saccharomyces cerevisiae*. Bobrowicz *et al*. [20] found that Arr1 (also known as Yap8 and which is a member of the YAP family that shares a conserved bZIP DNA-binding domain) confers resistance to arsenic by directly or indirectly regulating the expression of the plasma membrane pump Arr3 (also known as Acr3), another mechanism for arsenite detoxification of yeast in addition to the transporter gene, *YCF1 *[21]. Arr3 is 37% identical to a *Bacillus subtilis *putative arsenic-resistance protein and encodes a small (46 kilodalton (kDa)) efflux transporter that extrudes arsenite from the cytosol [22,23]. Ycf1, on the other hand, is an ATP-binding cassette protein that mediates uptake of glutathione-conjugates of AsIII into the vacuole [21,22]. Until recently, very little was known about arsenic-specific transcriptional regulation of detoxification genes. Wysocki *et al*. [24] found that Yap1 and Arr1 (called Yap8 in their paper) are not only required for arsenic resistance, but that Arr1 enhances the expression of Arr2 and Arr3 while Yap1 stimulates an antioxidant response to the metalloid. Menezes *et al*. [25], on the other hand, found that arsenite-induced expression of Arr2 and Arr3, as well as Ycf1, is likely to be regulated by both Arr1 (called Yap 8 in their paper) and Yap1.

Although Arr1 and Yap1 seem specifically suited for arsenic tolerance, the other seven YAP-family proteins are still worthy of investigation in light of the fact that each one regulates a specific set of genes involved in multidrug resistance with overlaps in downstream targets. One such interesting protein is Cad1 (Yap2). Although Yap1 and Cad1 are nearly identical in their DNA-binding domains, Yap1 controls a set of genes (including Ycf1) involved in detoxifying the effects of reactive oxygen species, whereas Cad1 controls genes that are over-represented for the function of stabilizing proteins in an oxidant environment [26]. However, Cad1 also has a role in cadmium resistance. As arsenic has metal properties, it is conceivable that Cad1 might play a greater part in arsenic tolerance and perhaps more so than the oxidative-stress response gene, *YAP1*.

Understanding the role of AP-1-like proteins (such as YAP family members) in metalloid tolerance was one of the goals in this study within the realm of the larger objective - using an integrative experimental and computational approach to combine gene expression and phenotypic profiles (multiplexed competitive growth assay) with existing high-throughput molecular interaction networks for yeast. As a consequence we uncovered the pathways that influence the recovery and detoxification of eukaryotic cells after exposure to arsenic. Networks were analyzed to identify particular network regions that showed significant changes in gene expression or systematic phenotype. For each data type, independent searches were performed against two networks: the network of yeast protein-protein and protein-DNA interactions, corresponding to signaling and regulatory effects (the regulatory network); and the network of all known biochemical reactions in yeast (the metabolic network). For the gene-expression analysis, we found several significant regions in the regulatory network, suggesting that Yap1 and Cad1 have an important role. However, no significant regions in the metabolic network were found. In order to test the functional significance of Yap1 and Cad1, we used targeted gene deletions of these and other genes, to test a specific model of transcriptional control of arsenic responses.

In contrast to the gene-expression data, the phenotypic profile analysis revealed no significant regions in the regulatory network, but two significant metabolic networks. Furthermore, we found that phenotypically sensitive pathways are upstream of differentially expressed ones, indicating that metabolic pathway associations can be discerned between phenotypic and transcriptional profiling. This is the first study to show a relationship between transcriptional and phenotypic profiles in the response to an environmental stress.

## Results and discussion

### Transcript profiling reveals that arsenic affects glutathione, methionine, sulfur, selenoamino-acid metabolism, cell communication and heat-shock response

Before gene-expression analysis of arsenic responses in *S. cerevisiae*, we performed a series of dose-response studies. We found that treatment of wild type cells with 100 μM and 1 mM AsIII had a negligible effect on growth, but that these cells still exhibited a pronounced transcriptional response (see Additional data files 1 and 2). Microarray analysis of biological replicates (four chips per replicate experiment) of the high-dose treated cells (1 mM AsIII) clustered extremely well together when using Treeview (see Materials and methods, and Additional data file 2). The lower dose time-course (100 μM AsIII) showed the beginning of gene-expression changes at 30 minutes, with the robust changes occurring at 2 hours, or one cell division (see Additional data file 2). The 2 hour, 100 μM dose clustered together with the 30 minute, 1 mM biological replicates and was in fact so similar to them that an experiment of one set of four chips for the 2 hour lower dose was deemed sufficient. Furthermore, when combining the three datasets (2 hour, 100 μM AsIII and each 30 minute, 1 mM AsIII replicate data) and using a 95% confidence interval (see Materials and methods) we found 271 genes that were not only statistically significant in at least 75% of the total data (9 out of 12 chips), but also that the direction and level of expression of these genes were similar between the datasets. The lower dose time-course also included a 4 hour treatment, or two cell divisions. This experiment demonstrated the greatest degree of variability, indicating either a cycling effect or the cell's return to homeostasis, which was further exemplified by a decrease in the transcriptional response (see Additional data file 2).

Genes were categorized by Kyoto Encyclopedia of Genes and Genomes (KEGG) pathway and Simplified Gene Ontology (biological process, cellular component and molecular function) (Table 1). In total, 829 genes out of 6,240 had significantly altered expression (see Materials and methods) in at least one experimental condition. The categories significantly enriched for differentially expressed genes in the KEGG pathways were glutathione, methionine, sulfur and selenoamino-acid metabolism, and in the Simplified Gene Ontology (biological process), cell communication and heat-shock response (Table 1).

### Network mapping of transcript profiling data finds a stress-response network involving transcriptional activation and protein degradation

**Figure 1:**
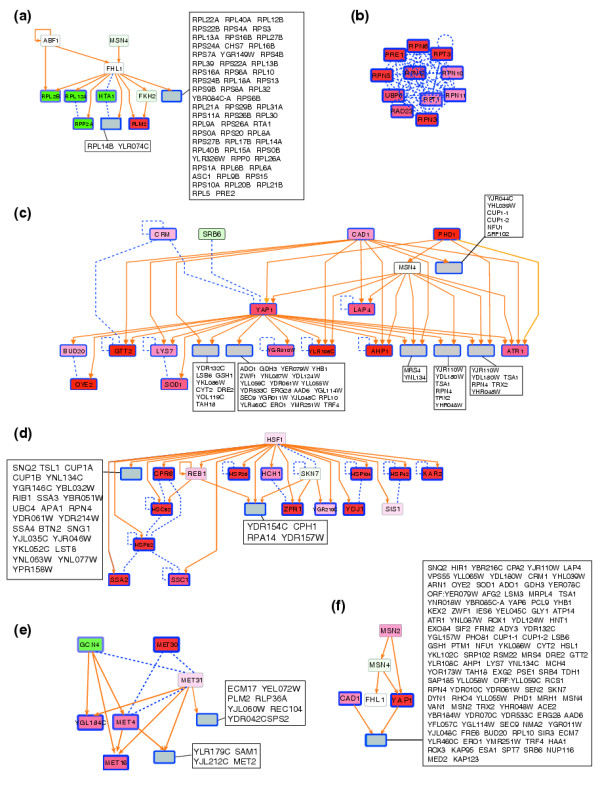
Arsenic-induced signaling and regulatory mechanisms involve transcriptional activators and the proteasome. **(a-d) **Significant network neighborhoods (*p *< 0.005) uncovered by the ActiveModules algorithm, with the search performed at depth 1 (all nodes in the network are the nearest neighbors of one central node): (a) *FHL1 *center; (b) *PRE1 *center and proteasome complex; (c) *YAP1 *and *CAD1 *centers; (d) *HSF1 *center. **(e) **An additional network centered on *MET31 *with functional relevance to the arsenic response, which, however, did not reach significance in this analysis, *p *< 0.11. **(f) **An overview of the network relationships between major arsenic-responsive transcription factors. Shades of red, induced; shades of green, repressed; blue boxed outline, significant expression; orange arrows, protein-DNA interaction; blue dashed lines, protein-protein interactions. The 2 h, 100 μM AsIII condition was used for the visual mappings. Many of the genes mapped to the network neighborhoods and displayed in this figure are boxed for the sake of clarity and space, but are mostly significantly differentially expressed.

We used the Cytoscape network visualization and modeling environment together with the ActiveModules network search plug-in to carry out a comprehensive search of the regulatory and metabolic networks [27,28]. The former consists of the complete yeast-interaction network of 20,985 interactions, in which 5,453 proteins are connected into circuits of protein-protein or protein-DNA interactions [29,30]. For each protein in this network, we defined a network neighborhood containing the protein and all its directly interacting partners. In the metabolic network, based on a reconstruction by Forster *et al*. [31] with 2,210 metabolic reactions and 584 metabolites, nodes represent individual reactions and edges represent metabolites. A shared metabolite links two reactions. We searched for sequences of related reactions governed by sensitive proteins (enzymes) in the phenotypic profiling data. To aid visualization, these sequences of reactions were combined to create metabolic pathways. We then identified the neighborhoods associated with significant changes in expression using the ActiveModules plug-in. This process resulted in the identification of seven significant neighborhoods in the regulatory network, centered on nodes Fhl1, Pre1, Yap1, Cad1, Hsf1, Msn2 and Msn4 (Figure 1). Together these neighborhoods narrow the significant data to 20% of the genes with the most significant changes in expression across one or more arsenic conditions (see Materials and methods and Additional data file 2). We did not find the emergence of any significant neighborhoods in the metabolic network.

The highest-scoring regulatory network neighborhood was defined by the transcription factor Fhl1 (Figure 1a). Its expression did not change significantly, but it was the highest-scoring node as judged by the significant expression changes observed for its surrounding neighborhood. Fhl1 controls a group of proteins important for nucleotide and RNA synthesis, as well as the synthesis and assembly of ribosomal proteins [32] which, from our data, are downregulated by arsenic exposure. Downregulation of ribosomal proteins in response to environmental stress has been reported previously [33,34], but to our knowledge this is the first association of Fhl1 as a key control element in this process. It seems likely that the repression of *de novo *protein synthesis in response to arsenic allows energy to be diverted to the increased expression of genes involved in stress responses and protection of the cell. One such pathway may involve sulfur metabolism, which leads to glutathione synthesis. In fact, included in Figure 1 is Met31 (Figure 1e), a transcriptional regulator of methionine metabolism, which interacts with Met4, an important activator of the sulfur-assimilation pathway that is probably involved in the glutathione-requiring detoxification process. While the differential expression of this neighborhood was not strictly significant according to ActiveModules (see Materials and methods), it has high biological relevance in light of the statistically significant alteration in expression categorized using KEGG pathways (Table 1).

Another high-scoring neighborhood comprises part of the proteasome protein complex (Figure 1b). The components of the proteasome are likely to be upregulated to meet the increased demand for protein degradation brought about by the binding of AsIII to the sulfhydryl groups on proteins and/or glutathione that subsequently interfere with numerous enzyme systems such as cellular respiration [7,15]. In this paper, we will propose that this occurs through indirect oxidative stress as a result of the depletion of glutathione.

### The role of transcription factors Yap1 and Cad1 and the metalloid stress response

Many of the central proteins in the significant neighborhoods uncovered by ActiveModules were transcription factors (Figure 1a,c-f). Although some of these proteins were not differentially expressed themselves, they were still high-scoring nodes because of the highly significant expression of their targets. This is also important to keep in mind as we discuss later which genes might be sensitive to arsenic, but not necessarily differentially expressed, and why many genes that are differentially expressed do not display sensitive phenotypes when deleted.

Transcription factors Msn2, Yap1, Msn4, Cad1 and Hsf1 were the central proteins for many of the significant neighborhoods found (Figure 1c,d,f). Together with several genes previously implicated in oxidative-stress responses, these neighborhoods compose a stress-response network [24,26,35-39]. Of particular interest are Yap1 and Cad1, because of the high number of shared downstream targets (Figure 1c,f).

When overexpressed, Yap1 confers resistance to several toxic agents, and Yap1 mutants are hypersensitive to oxidants [33,40-44]. Conversely, Cad1 responds strongly to cadmium, but not to hydrogen peroxide (H_2_O_2_) [26,35]. Following arsenic exposure, Yap1 is induced at least fourfold, with many of its downstream targets showing high levels of induction (see Additional data file 3). Several of its targets are among the most highly upregulated genes (as high as 178-fold for *OYE3 *(encoding a NADPH dehydrogenase)). Moreover, Yap1 regulates *GSH1*, which encodes γ-glutamylcysteine synthetase (an enzyme involved in the biosynthesis of antioxidant glutathione), *TRX2 *(the antioxidant thioredoxin), *GLR1 *(glutathione reductase) and drug-efflux pumps *ATR1 *and *FLR1 *[35,45-50]. It should be noted that *GSH1 *and *ATR1 *are examples of several genes also targeted by Cad1. All of these specified Yap1 targets are induced after arsenic exposure, recapitulating the toxicant's role as a likely oxidant. During the course of this work, Wysocki *et al*. [24] also implicated Yap1 in arsenic tolerance.

**Figure 2:**
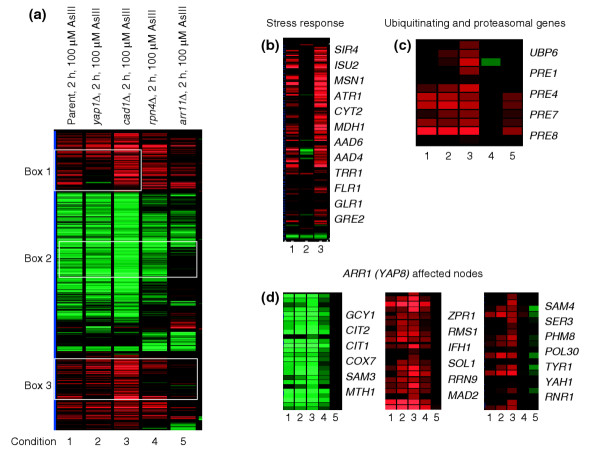
Yap1 but not Cad1 is important for mediating the cell's adaptation to arsenic. **(a) **Self-organized heat map (dendograms were removed and boxes 1-3 indicate specific clusters) of 6,172 genes selected from the various indicated conditions. AsIII-treated parent wild type strain with normalized data values that are greater or less than those in condition(s) knocked-out Yap1, Cad1, Rpn4, or Arr1 treated with AsIII, by a factor of twofold. All knockouts tested revealed altered profiles compared to the wild type, except for *cad1Δ*. **(b) ***yap1Δ *(condition 2) loses induced expression of stress response genes found in box 1, such as *SIR4*, *ISU2*, *MSN1*, *ATR1*, *CYT2*, *MDH1*, *AAD6*, *AAD4*, *TRR1*, *FLR1*, *GLR1 *and *GRE2*. **(c) ***rpn4Δ *(condition 4) loses induced expression of ubiquitinating and proteasomal genes found in box 3 - *UBP6*, *PRE8*, *PRE4*, *PRE7 *and *PRE1*. **(d) ***arr1Δ *(condition 5) loses repressed expression of sulfur amino-acid metabolism gene *SAM3 *and glutamate biosynthesis gene *CIT2*, among others (box 2). *arr1Δ *also loses induced expression of serine biosynthesis gene *SER3*, sulfur amino-acid metabolism gene *SAM4*, cell-cycle regulator *ZPR1*, spindle-checkpoint subunit *MAD2*, ribonucleotide reductase *RNR1*and RNA polymerase I transcription factor *RRN9*, to name a few (box 3). Red, induced; green, repressed. For a comprehensive list of genes affected in all knockout experiments, see the Additional data files with the online version of this paper.

As Cad1 and Yap1 share many downstream targets, the genes defined by these transcription factors are very similar. To determine which transcription factor is playing the most active role in the high level of differential expression for this group (see Figure 1c,f), we tested the roles of both activators by treatment of *yap1Δ *and *cad1Δ *deletion strains with 100 μM AsIII for 2 hours (Additional data file 4). Surprisingly, we did not find that Cad1 was involved in regulation in response to arsenic-mediated stress. The *yap1Δ *strain was not only sensitive to AsIII by phenotypic profiling (Additional data file 5) but also defective in the induction of several downstream enzymes with antioxidant properties (Figure 2a,b). Conversely, the *cad1Δ *strain displayed an almost identical profile to wild type, eliminating it as a strong factor in the arsenic response (Figure 2a,b). A list of arsenic-mediated genes with at least a twofold difference in expression compared to wild type for *yap1Δ *and *cad1Δ *is provided (Additional data files 6 and 7). These were generated using Rosetta Resolver with a *p*-value less than 0.001 (see Materials and methods for more detail). Also, Additional data files 8 and 9 contain tables of genes failing to be induced or repressed (or showing such a decrease in expression that they no longer make significantly expressed gene lists) in the *yap1Δ *and *cad1Δ *experiments, compared to the parent experiment, after treatment with 100 μM AsIII for 2 hours. These are lists of genes that would be potentially regulated by Yap1 and Cad1 in the presence of arsenic.

### The proteasome responds to arsenic, and Rpn4 mediates a transcriptional role

**Figure 3:**
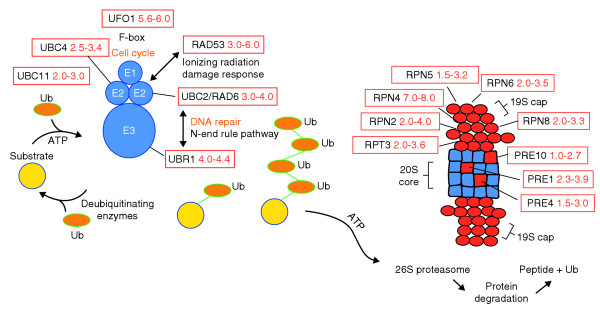
The ubiquitin (Ub) and proteasome system responds to arsenic-mediated toxicity. *S. cerevisiae *ubiquitin and proteasome pathways show differential expression in a number of key genes, including that for the proteasomal activator *RPN4*. Induction is denoted by red boxes with fold-change ranges representing the 2 h, 100 μM AsIII and 0.5 h, 1 mM AsIII experiments, respectively.

Treatment of yeast with as little as 100 μM AsIII for 2 hours resulted in the induction of at least 14 ubiquitin-related and proteasome gene products (Figure 1b and Figure 3). The eukaryotic proteasome consists of a 20S protease core and a 19S regulator complex, which includes six AAA-ATPases known as regulatory particle triple-A proteins (RPT1-6p) [51,52]. Proteins are targeted for degradation by the proteasome via the covalent attachment of ubiquitin to a lysine side chain on the target protein (Figure 3). Conjugating enzymes then function together with ubiquitin-ligase enzymes to adhere to the target protein, and are tailored to carry out specific protein degradation in DNA repair, growth control, cell-cycle regulation, receptor function and stress response, to name a few [53,54]. The apparent importance of Yap1 in response to possible oxidative damage by arsenic indicated a potential role for Rpn4 (induced eightfold, Figure 3). This is a 19S proteasome cap subunit, which also acts as a transcriptional activator of the ubiquitin-proteasome pathway and a variety of base-excision and nucleotide-excision DNA repair genes [34,55,56].

Rpn4 is required for tolerance to cytotoxic compounds and may regulate multidrug resistance via the proteasome [57]. Moreover, Owsianik *et al*. [57] identified an YRE (Yap-response element) site present in the *RPN4 *promoter. This YRE was found to be functional and important for the transactivation of *RPN4 *by Yap1 in response to oxidative compounds, such as H_2_O_2_. However, we also located the Rpn4-binding sequence, TTTTGCCACC, 47 bases distant from the open reading frame (ORF) of *YAP1*, indicating that Yap1 not only activates Rpn4, but that Rpn4 may in fact activate Yap1 [58]. In support of this hypothesis we found that relative to wild type, the level of Yap1 induction was lower in the *rpn4Δ *strain under arsenic stress conditions, whereas Rpn4 was equally induced in the *yap1Δ *strain (Additional data file 10).

With respect to wild type, the profile of *rpn4Δ *after treatment with arsenic was the most dramatically altered, save for *arr1Δ *(Figure 2 and Additional data files 11 and 12). These data suggest that arsenic modification of sulfhydryl groups on proteins leads to protein inactivation and therefore degradation via the 26S proteasome. Another scenario is that the proteasome, and/or its proteases, is sensitive to arsenic-related events, leading to dysfunctional protein turnover and an increased requirement for 26S proteasome subunits. A similar idea was proposed for the direct methylating agent, methylmethane sulfonate [34].

### *ARR1* transcriptional responses

Arr1 is structurally related to Yap1 and Cad1 [20,24]. However, little is known about how Arr1 may be involved in oxidative stress and/or multidrug resistance. Furthermore, Arr1 is not well represented by the interactions present in the yeast regulatory network. However, studies by Bobrowicz *et al*. [20,59] show that the transcriptional activation of Arr3 requires the presence of the Arr1 gene product. Moreover, a report by Bouganim *et al*. [60] supports our finding that Yap1 also is important for arsenic resistance. They show that overproduction of Yap1 blocks the ability of Arr1 to fully activate Arr3 expression at high doses of arsenite, suggesting that Yap1 can compete for binding to the promoter of the Arr1 target gene, *ARR3*. While this paper was being written, Tamas and co-workers [24] showed that Arr1 transcriptionally controls Arr2 and Arr3 expression from a plasmid containing their promoters fused to the *lacZ *gene and measuring β-galactosidase activities. This was done by growing the cells for 20 hours with a low dose of metalloid and spiking the concentration to 1 mM AsIII for the last 2 hours of incubation. These experiments showed that *ARR1 *deletion resulted in complete loss of Arr3-*lacZ *induction, whereas *YAP1 *deletion did not significantly affect induction. Similar results were obtained for the Arr2-*lacZ *induction assay and the authors concluded that Yap1 has a role in metalloid-dependent activation of oxidative stress response genes, whereas the main function of Arr1 seems linked to the control of Arr2 and Arr3. Interestingly, this study was shortly followed by another from Menezes *et al*. [25] which found contrasting results when looking at mRNA and Northern-blot analysis. In this study, the induction of Arr2 and Arr3, after treatment with 2 mM AsIII for up to 90 minutes, did not occur in either the *ARR1*-deleted strain or the *YAP1*-deleted strain. These authors conclude that the requirement for both *YAP1 *and *ARR1 *is vital to yeast in the function of regulating and inducing genes important for arsenic detoxification. Finally, transcription profiling experiments presented here show that the arsenic transport proteins Arr2 and Arr3 are still expressed (2.9-fold induction for Arr2 and 1.8-fold for Arr3, respectively) in the *ARR1 *mutant, but show defective induction in the *yap1Δ *strain treated in parallel (Additional data files 4 and 10). These results indicate that Yap1 may control Arr2 and Arr3 when yeast is subjected to 100 μM AsIII for 2 hours.

Our results and those of Menezes *et al*. [25], in contrast to the results of Tamas and colleagues [24], might be explained by the following. Our and Menezes *et al.*'s studies looked at genes in the normal chromosome context rather than genes ectopically expressed from a plasmid; in addition, in our study, we treated the yeast with 100 μM AsIII while Wysocki *et al*. [24] started with a low dose, but spiked the concentration to 1 mM AsIII in the last 2 hours of incubation. However, Menezes *et al*. [25] used an even higher dose (2 mM AsIII for a time-course ending at 90 minutes) and obtained more similar results to ours, with the exception that their Northern-blot analysis, which can sometimes miss relatively small changes, indicated an apparent lack of induction of *ARR2 *or *ARR3 *in either the *ARR1*- or *YAP1*-deleted strains. Taken together, these data indicate that both *ARR1 *and *YAP1 *are important genes involved in the process of arsenite detoxification in the yeast cell, but because of the different strains and treatment protocols used between these three studies, further experiments are warranted to resolve the differences.

Other interesting results from our transcription profiling of the *arr1Δ *and parent strains after arsenic treatment (Figure 2a,d and Additional data files 13 and 14), included large differences in expression as a whole and in particular the inability of *arr1Δ *to induce serine biosynthesis-related genes such as *SER3*, and sulfur and methionine amino-acid metabolism genes including *SAM4*. Conversely, *arr1Δ *failed to repress *SAM3*, as well as *CIT2*, a glutamate biosynthesis gene, when compared to the parent profile.

These observations indicate that Arr1 may regulate sulfur-assimilation enzymes that are necessary for arsenic detoxification. This is particularly interesting considering that the ActiveModules algorithm identified the node Met31 (Figure 1e), the transcriptional regulator of methionine metabolism which interacts with Met4, an important activator of the sulfur-assimilation pathway that is likely to be involved in the glutathione-requiring detoxification process. Sulfur metabolism was also a functional category in the Simplified Gene Ontology found to be significantly enriched by the hypergeometric statistical test (see Materials and methods) (Table 1). Furthermore, phenotypic profiling results discussed later show the importance of serine and glutamate metabolism in the sensitivity response to arsenic. Lastly, it is important to note that *arr1Δ *also displays loss of expression of a number of ubiquitin-proteasome-related gene products, sharing similar expression patterns with *rpn4Δ *(Additional data files 13 and 14) and suggesting that it may have a role in protein degradation as well.

### Arsenic treatment stimulates cysteine and glutathione biosynthesis and leads to indirect oxidative stress

Our arsenic-treatment experiments revealed the strong induction of over 20 enzymes in the KEGG sulfur amino acid and glutathione biosynthesis pathways (Table 1). This is consistent with the hypothesis that glutathione acts as a first line of defense against arsenic by sequestering and forming complexes with the toxic metalloid [21].

**Figure 4:**
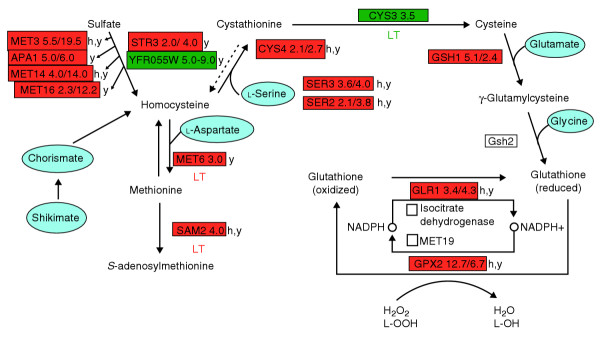
Gene-expression profiling links sulfur assimilation, methionine and glutathione pathways. Selected genes in these pathways are represented as red for induced (2 h, 100 μM AsIII and 0.5 h, 1 mM AsIII, respectively) and green for repressed. Genes in white boxes are not differentially expressed. The pathways in the blue ovals are upstream of methionine, cysteine and glutathione, and are sensitive to arsenic. The downstream pathways employ numerous redundant enzymes that are differentially expressed, but are not sensitive. LT, late time-point, 4 h, 100 μM AsIII experiment; h, human; y, yeast.

Dormer *et al*. [61] showed that *GSH1 *induction by cadmium is dependent on the presence of Met4, Met31, Met32 and Cbf1 in the transcriptional complex of MET genes. Met4 and Met32 are also differentially expressed in response to arsenic and interact with Met31, which defines a network neighborhood as shown in Figure 1e. The biological impact of the sulfur-related stress response was further exemplified by comparisons of our arsenic profiles to H_2_O_2 _profiles (400 μM H_2_O_2_) from Causton *et al*. [62] (Table 2). Although we found many expected similarities between arsenic and H_2_O_2 _gene-expression profiles in regard to oxidative-stress response genes, sulfur and methionine metabolism genes, in response to H_2_O_2_, were either repressed or did not change (Table 2). Furthermore, a study by Fauchon *et al*. [63] showed that yeast cells treated for 1 hour with 1 mM of the metal Cd^2+^, responded by converting most of the sulfur assimilated by the cells into glutathione, thus reducing the availability of sulfur for protein synthesis. Our arsenic profile showed a similar response to the sulfur-assimilation profile seen with Cd^2+ ^(Table 2). As a consequence, arsenic may be conferring indirect rather than direct oxidative stress mediated by the depletion of glutathione, thus inhibiting the breakdown of increasing amounts of H_2_O_2 _by glutathione peroxidase (*GPX2*, up 13-fold) (Figure 4) [21,64].

### Phenotypic profiling defines arsenic-sensitive strains and maps to the metabolic network

To identify genes and pathways that confer sensitivity to arsenic, we identified deletion mutants with increased sensitivity to growth inhibition using a deletion mutant library of nonessential genes (4,650 homozygous diploid strains) [65,66]. Each strain contains two unique 20-bp sequences (UPTAG and DOWNTAG) enabling their growth to be analyzed *en masse *and the fitness contribution of each gene to be quantitatively assayed by hybridization to high-density oligonucleotide arrays. The top 50 sensitive deletion strains included: *THR4*, *SER1*, *SER2*, *CPA2*, *CPA1*, *HOM2*, *HOM3*, *HOM6*, *ARG1*, *YAP1*, *CDC26*, *ARR3*, *CIN2*, *ARO1*, *ARO2 *and *ARO7*. A listing of the rank order for all sensitivities is available (Additional data file 5).

Only 10% of the top 50 sensitive mutant strains were significantly differentially expressed in the transcript profile. This lack of direct correlation between gene expression and fitness data is consistent with data from our own and other laboratories [2,4,65]. At least three factors may contribute to this discrepancy. First, some highly expressed genes when deleted are nonviable (around 1,000 genes) and are therefore unable to be scored for fitness. Some examples of highly expressed, yet nonviable, genes under arsenic stress are *ERO1 *(7- to 10-fold induced), *HCA4 *(5- to 9-fold induced), and *DCP1 *(9- to 22-fold induced). Second, there are redundant pathways mediated by multiple genes, such that deletion of one does not lead to sensitivity. *OYE2*, *OYE3*, and a large number of reductases fall into this category. Finally, gene products that do not change significantly, mediate important biological responses and thus when deleted could sensitize the cell to a specific stressor. *ARO1*, *ARO2*, *THR4 *and *HOM2 *are examples of genes that are not differentially expressed but are very sensitive to arsenic.

**Figure 5:**
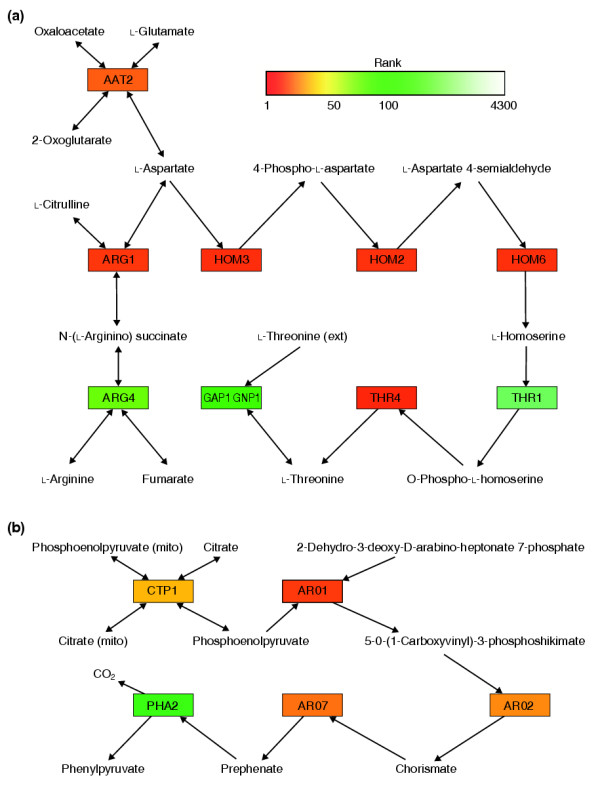
LinearActivePaths analysis finds that virtually all genes in active metabolic networks confer sensitivity to arsenic when deleted. **(a) **Serine, threonine, glutamate amino-acid synthetic pathways; **(b) **the shikimate pathway. The paths that compose these networks all have individual *p*-values of < 0.05. The coloration for these figures is based on red for any gene ranked in the top 50 significant genes, yellow for 51-100, and green for >101.

Like the gene-expression data, the phenotypic data was subjected to searches performed against the regulatory network of yeast protein-protein and protein-DNA interactions as well as the metabolic network of all known biochemical reactions in yeast. Unlike the transcription profile, the phenotypic data analysis revealed no significant regions in the regulatory network, but did map to two statistically significant metabolic networks. The first significant pathway was amino acid synthesis/degradation with the terminal products being L-threonine and L-homoserine, beginning with precursors such as L-arginine, fumarate and oxaloacetate (Figure 5a). These products function in serine, threonine and glutamate metabolism. The second network indicated the importance of the shikimate pathway, which is essential for the production of aromatic compounds in plants, bacteria and fungi (Figure 5b). The shikimate pathway operates in the cytosol of yeast and utilizes phosphoenol pyruvate and erythrose 4-phosphate to produce chorismate through seven catalytic steps. It is a pathway with multiple branches, with chorismate representing the main branch point, and various branches giving rise to many end products. Interestingly, chorismate is also used for the production of ubiquinone, *p*-aminobenzoic acid (PABA) and folates, which are donors to homocysteine [67-69].

### Relationship between gene-expression and phenotypic profiles

**Figure 6:**
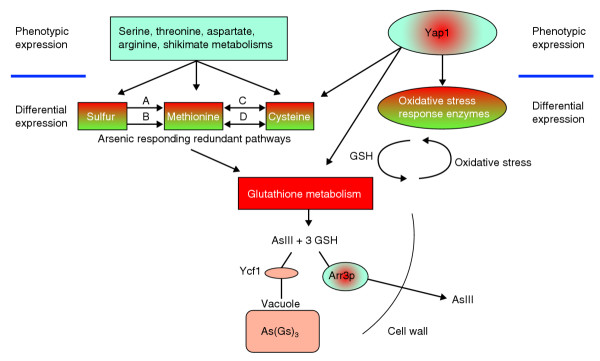
Global model of the arsenic response: combining phenotypic data with gene-expression profiles reveals synergistic pathways leading to yeast detoxification mechanisms. Serine, threonine, aspartate and arginine, as well as shikimate metabolisms, in light blue, represent pathways that are judged as sensitive by phenotypic profiling. Yap1, colored light blue and red, is an example of a transcription factor that is both sensitive and confers induced gene expression. Deletion analysis confirms its role in arsenic-mediated control of the stress response. Red and green represent pathways or genes that are differentially expressed but not sensitive by phenotypic profiling. This schematic diagram demonstrates how the deletion of an individual gene leads to a change in sensitivity if the protein product of that gene is important in a biological process for adaptation to arsenic. On the other hand, expression profiling shows the end product of the cell's response to arsenic. Many of these downstream targets share redundant functions and are not vulnerable in the phenotypic profiling. The expression changes lead to the cell's response to indirect oxidative stress and mechanisms for detoxification. The arrows A, B, C and D represent the multiple branchpoints between redundant pathways. Note that the transport protein, Arr3, which extrudes AsIII out of the cell, is both sensitive and highly differentially expressed.

Combining transcript profiling and phenotypic profiling provides deeper insights into the biology of arsenic responses. Until now there has been a lack of correlation between the differential expression of genes and sensitivity of deletion mutants [2,4,65] and this was the case in the present study. However, by mapping each dataset to the regulatory and metabolic networks, we have uncovered the likely reason for this lack of congruence. Our data show that many of the most sensitive genes (Additional data file 5; top 50 ranks) are involved in serine and threonine metabolism, glutamate, aspartate and arginine metabolism, or shikimate metabolism, which are pathways upstream of the differentially expressed sulfur, methionine and homocysteine metabolic pathways, respectively. These downstream pathways are important for the conversion to glutathione, necessary for the cell's defense from arsenic (Figures 4, 5a, 6 and Table 1). This overlap of sensitive upstream pathways and differentially expressed downstream pathways provides the link between transcriptional and phenotypic profiling data (Figures 4 and 6).

Thus, we believe our work shows that the deletion of an individual gene can lead to a change in sensitivity to an agent only if the protein product of that gene is important for some process (for example, amino-acid synthesis or a transcription factor required for the increased expression of genes needed to protect against the agent). On the other hand, expression profiling shows the end product of the cell's response to arsenic. Therefore, an agent such as arsenic might cause a transcription factor (Yap1, for example) to increase the expression of as many as 50 genes, 20 of which might help to protect against the agent. However, deletion of any of the 50 would not be expected to have an effect on the response to arsenic. The effect of gene deletion would be on the transcription factor itself (whose expression might not be affected by the agent). Thus, in the case of arsenic exposure, we conclude that phenotypic profiling interrogates genes upstream of the genes that ultimately protect against arsenic toxicity and that the downstream targets that demonstrate differential expression probably share redundant functions and are not vulnerable in the phenotypic profiling (Figure 6).

## Conclusions

Systems biology represents an important set of methods for understanding stress responses to environmental toxicants, such as arsenic. In this study we have catalogued the centers of activity associated with arsenic exposure in yeast, identifying the key neighborhoods of activity in the regulatory and metabolic networks using the visualization tools and algorithms in Cytoscape. The transcriptional profile mapped to the regulatory network, revealing several important nodes (Fhl1, Msn2, Msn4, Yap1, Cad1, Pre1, Hsf1 and Met31) as centers of arsenic-induced activity. From these results we can conclude that arsenic detoxification in yeast focuses around: nucleotide and RNA synthesis; methionine metabolism and sulfur assimilation; protein degradation; and transcriptional regulation by proteins that form a stress-response network. In summary, protein synthesis in response to arsenic allows energy to be diverted toward the genes channeling sulfur into glutathione, which then leads to indirect oxidative stress by depleting glutathione pools and alters protein turnover. These processes require regulation by transcription factors, the understanding of which we refined by analysis of specific knockout strains. Our experiments, in fact, confirmed that the transcription factors Yap1, Arr1 and Rpn4 strongly mediate the cell's adaptation to arsenic-induced stress but that Cad1 has negligible impact. Finally, contrary to the gene-expression analyses, the phenotypic profiling data mapped to the metabolic network. The two significant metabolic networks unveiled were shikimate and serine, threonine and glutamate biosynthesis. Our goal was to integrate the computational identification of these important pathways found via transcript and phenotypic profiling by regulatory and metabolic network mapping. In doing so, we have shown that genes that confer sensitivity to arsenic are in pathways that are upstream of the genes that are transcriptionally controlled by arsenic and share redundant functions.

## Materials and methods

### Strains, media and growth conditions

*S. cerevisiae *strain BY4741 (*MAT***a**, *his3Δ*, *leu2Δ0*, *met15Δ0*, *uraΔ0*) was used and grown in synthetic complete medium at 30°C. Cells were grown to a density of 1 × 10^7 ^cells per ml. Cultures were split into two; NaAsO_2_ (100 μM and 1 mM in two biological repeats) was added to one culture, and both were incubated at 30°C for 0.5, 2 or 4 h. Cells were pelleted and washed in distilled water before RNA extraction. Deletion strains (*yap1Δ*, *cad1Δ*, *arr1Δ *and *rpn4Δ*) of the same background were obtained from Research Genetics, confirmed and treated the same way, for 2 h and 100 μM NaAsO_2_.

### RNA extraction

For the cDNA hybridization experiments, total RNA was isolated using an acid-phenol method. Pellets were resuspended in 4 ml lysis buffer (10 mM Tris-HCL pH 7.5, 10 mM EDTA, 0.5% SDS). Four milliliters of acid (water-saturated, low pH) phenol was added followed by vortexing. The lysing cell solutions were incubated at 65°C for 1 h with occasional vigorous vortexing and then placed on ice for 10 min before centrifuging at 4°C for 10 min. The aqueous layers were re-extracted with phenol (room temperature, no incubation) and extracted once with chloroform. Sodium acetate was then added to 0.3 M with 2 volumes of absolute ethanol, placed at -20°C for 30 min, and then spun. Pellets were washed two or three times with 70% ethanol followed by Qiagen Poly(A)^+ ^RNA purification with the Oligotex oligo (dT) selection step. Total RNA for the specific knockout strains and parent experiment was isolated by enzymatic reaction, following the RNeasy yeast protocol (Qiagen).

### Microarray hybridizations and analyses

A cDNA yeast chip, developed in-house at National Institute of Environmental Health Sciences (NIEHS), was used for gene-expression profiling experiments. A complete listing of the ORFs on this chip is available at [70]. cDNA microarray chips were prepared as previously described [71,72]. The cDNA was spotted as described [73]. Each poly(A) RNA sample (2 μg) was labeled with Cy3- or Cy5-conjugated dUTP (Amersham) by a reverse transcription reaction using the reverse transcriptase SuperScript (Invitrogen), and the primer oligo(dT) (Amersham). The hybridizations and analysis were performed as described Hewitt *et al*. [74] except that genes having normalized ratio intensity values outside of a 95% confidence interval were considered significantly differentially expressed. Lists of differentially expressed genes were deposited into the NIEHS MAPS database [75]. Genes that were differentially expressed in at least three of the four replicate experiments were compiled and subsequently clustered using the Cluster/Treeview software [76]. GeneSpring (Silicon Genetics) and Cytoscape [28] were used to further analyze and visualize the data.

The knockout experiments were conducted on an Agilent yeast oligo array platform. Samples of 10 μg total RNA were labeled using the Agilent fluorescent direct label kit protocol and hybridizations were performed for 16 h in a rotating hybridization oven using the Agilent 60-mer oligo microarray-processing protocol. Slides were washed as indicated and scanned with an Agilent scanner. Data was gathered using the Agilent feature extraction software, using defaults for all parameters, save the ratio terms. To account for the use of the direct label protocol, error terms were changed to: Cy5 multiplicative error = 0.15; Cy3 multiplicative error = 0.25; Cy5 additive error = 20; Cy3 additive error = 20.

GEML files and images were exported from the Agilent feature extraction software and deposited into Rosetta Resolver (version 3.2, build 3.2.2.0.33) (Rosetta Biosoftware). Two arrays for each sample pair, including a fluor reversal, were combined into ratio experiments in Rosetta Resolver. Intensity plots were generated for each ratio experiment and genes were considered 'signature genes' if the *p*-value was less than 0.001. *p*-values were calculated using the Rosetta Resolver error model with Agilent error terms. The signature genes were analyzed with GeneSpring. The entire in-house and Agilent-based dataset is available in the Additional data files.

### Ontology enrichment

Genes have previously been categorized into various ontologies and pathways. If a particular pathway is enriched for genes that are significantly expressed in response to a process, we conclude that the pathway is likely to be involved in this process. In total, 829 genes out of 6,240 had a significant alteration in expression in at least one experimental condition. Along with the size of each functional category, a statistical measure for the significance of the enrichment was calculated by using a hypergeometric test. The level of significance for this test was determined using the Bonferroni correction, where the α value was set at 0.05 and the number of tests conducted for KEGG pathway and Simplified Gene Ontology (biological process) were 27 and 11, respectively.

### Network searches

The ActiveModules algorithm was used to identify neighborhoods in the regulatory network corresponding to significant levels of differential expression. In this search, if a protein has many neighbors, it is likely that at random a few will show significant changes in expression and these could be selected as a significant sub-network. Neighborhood scoring is a method we used to correct for this bias. In this scheme, a significant sub-network must contain either all or none of the neighbors of each protein. The significance then represents an aggregate over all neighbors of a protein. This prevents the biased selection of a few top-scoring proteins out of a large neighborhood in the search for significant sub-networks. For an in-depth description of this algorithm see Ideker *et al*. [1].

In defining the network used in the metabolic analysis, edges corresponding to metabolites linking more than 175 reactions were eliminated. This excludes metabolic cofactors such as ATP, NADH and H_2_O from the search. Scores for each ORF were generated by mapping the fitness significance value to a Z-score. To assign scores to the individual reactions, Förster's mapping from ORF to reaction was used to generate a list of ORFs for each reaction. The Z-scores of these ORFs were then aggregated into a single score for that reaction using the following equation:



We used a dynamic programming algorithm adapted from Kelley *et al*. [77] to identify high-scoring paths in this network. Briefly, the highest-scoring path of length (*n*) ending at each node is determined by combining the scores of the individual node and the highest-scoring path of length (*n *- 1) ending at a neighbor node using the following formula:



Since a node with many neighbors is more likely to belong to a high-scoring path by random chance, the score of the neighboring path is corrected against the extreme-value statistic with the number of observations equal to the number of neighbors.

The significances of the top-scoring networks were determined by comparison to a distribution of the top-scoring networks from random data (reaction scores randomized with respect to the nodes of the network). After running the path finding/scoring algorithm, the score of the single highest-scoring path was added to the null distribution. This process was repeated for 10,000 interactions. This null distribution was then used to determine an empirical *p*-value, which represents the null hypothesis that there is no significant correlation between the topology of the metabolic network and the assignment of significance values to nodes in that network.

### Specific deletion experiment filter on fold-change comparisons

The intensity plots were generated from each experiment in Rosetta Resolver. A gene was considered a signature gene if the *p*-value was less than 0.001 and if the fold-change value was greater than or equal to twofold. Signature genes were then broadcasted on the intensity plot and exported as text files. Lists were imported into GeneSpring. The 'Filter on Fold Change' function was used to compare the parent control vs. parent AsIII experiment with each deletion (AsIII) experiment. The gene list selected for each filter on fold change analysis was a combination of the parent signature gene list and the signature gene list of the AsIII-treated deletion being analyzed at the time. For example, if the comparison was being done between parent (AsIII-treated) and Yap1 (AsIII-treated), the list used in the analysis was the combination of the parent signature genes and the Yap1 signature genes. The filter on fold change function reports genes that were selected from the one condition (parent) that had normalized data values that were greater or less than those in the other condition (deletion under investigation) by a factor of twofold. Each resulting gene list was saved. All the resulting gene lists were combined and an annotated gene list was exported for use in Eisen's Cluster/Treeview package (described earlier). The format of the exported data was the natural log. The gene tree generated for the paper was generated in GeneSpring. Each filter on fold change was saved as an annotated gene list.

### Generation of specific deletion experiment 'minus' lists

Signature gene lists were generated in Rosetta Resolver from intensity plots as described above. Each signature gene list was saved as a 'Bioset' in Resolver. The parent Bioset was compared to each deletion Bioset using the 'Minus' function. This function finds those members in Bioset group 1 (parent) that do not exist in Bioset group 2 (deletion). Each of the resulting lists was saved as a new Bioset. The new 'minus' Bioset was broadcasted on its corresponding intensity plot and exported as a text file. This was repeated for each experiment with fine-tuning of the data using GeneSpring.

### Phenotypic profiling

Homozygous diploid deletion strains and pooling of the strains were done as described [66]. Aliquots were grown until logarithmic phase, diluted to OD_600 _0.05-0.1, split into tubes and treated with arsenic for 1-2 h at 1 mM, 2 mM and 5 mM concentrations. Similar responses were observed at each concentration, so the results were pooled. These cultures and a mock-treated sample were maintained in logarithmic phase growth by periodic dilution for 16-18 h. UPTAG and DOWNTAG sequences were separately amplified from genomic DNA of the drug and mock-treated samples by PCR using biotin-labeled primers as described previously [66]. The amplification products were combined and hybridized to Tags3 arrays (Affymetrix). Procedures for PCR amplification, hybridization and scanning were done as described [66], and according to the manufacturer's recommendation when applicable. The images were quantified by using the Affymetrix Microarray Suite software. UPTAG and DOWNTAG values were separately normalized, ratioed (treated sample signal/control) and filtered for intensities above background [78].

## Additional data files

The following additional data files are available with the online version of this article and at [79]. Additional data file 1 shows the dose-response curve of *S. cerevisiae *strain BY4741 (*MAT***a**, *his3Δ*, *leu2Δ0*, *met15Δ0*, *uraΔ0*) grown in synthetic complete medium at 30°C after treatment with arsenic. Treatment with 1 mM, 2 mM and 5 mM AsIII resulted in a negligible effect on growth (after 18 h) and survival (1 h treatment followed by plating and colony formation counting), but still exhibited a pronounced transcriptional response (see Additional data file 2). Additional data file 2 contains a figure showing all genes found to be significant by MAPS analysis (see Materials and methods) which were compiled across the four arrays, averaged and subsequently clustered with Cluster/Treeview software (Eisen *et al. *[76]). The dendogram highlighted in pink depicts the zoomed in region shown to the right of the entire tree. Genes in red are induced and genes in green are repressed. A table depicts the numbers of genes changing in each experiment at both the 95% and 99% confidence intervals (see Materials and methods). Additional data file 3 contains the primary raw cDNA data from all the experiments. Additional data file 4 contains the primary raw data for all the deletion strain experiments. Additional data file 5 contains the sensitivity (phenotypic profiling) data ranked on the basis of four experiments, 1 mM (2x), 2 mM and 5 mM AsIII, and assigned a new uniform distribution of *p*-values. Every gene in this table has a percentile rank. In the case that there was slow growth in the wild type, then a default value of 0.5 was assigned. The rankings on this table were used for the metabolic networking. Additional data files 6 and 7 contain data produced by applying the 'Filter on Fold Change' function in GeneSpring after importing the significant gene lists generated using Rosetta Resolver with a *p*-value less than 0.001 (see Materials and methods for more detail). The control parent vs. parent experiment (100 μM AsIII for 2 h) was compared with the *yap1Δ *(Additional data file 6) and *cad1Δ *(Additional data file 7) profiling experiments treated in parallel (for details see Materials and methods). Additional data files 8 and 9 contain tables of genes ('Minus' lists) that failed to be induced or repressed (or showed such a decrease in expression that they no longer make significantly expressed gene lists), compared to the parent experiment, in the *yap1Δ *(Additional data file 8) and *cad1Δ *(Additional data file 9) experiments after treatment with 100 μM AsIII for 2 h. Additional data file 10 contains a figure showing that Yap1 is likely to regulate Arr2 and Arr3 after 2 h 100 μM AsIII but that it does not regulate Rpn4 under arsenic-induced stress. The self-organized heat map labeling and conditions in this figure are the same as for Figure 2. (a) The Yap1 knockout strain fails completely to induce Arr2 (0.834 average fold-change) whereas the Arr1 knock-out induces Arr2 (2.90 average fold-change). (b) The Arr1 knockout induction is more elevated compared to the Yap1 knock-out (1.8 and 1.1 average fold-change, respectively). (c) Yap1 is induced 2.7 fold in the Rpn4 knock-out. (d) The wild type parent strain shows an averaged induction of 4.7 fold. (e) Rpn4 is induced 3.7 fold in the Yap1 knock-out compared to 4.1 fold induction in the wild type parent strain. In the presence of arsenic, Yap1 does not appear to regulate Rpn4. Additional data file 11, as explained for Additional data files 6 and 7, compares the control parent vs. parent experiment (100 μM AsIII for 2 h) to the *rpn4Δ *profiling experiment treated in parallel. Additional data 12 contains a table of genes ('Minus' list) that fail to be induced or repressed, compared to the parent experiment, in the *rpn4Δ *experiment after treatment with 100 μM AsIII for 2 h. Additional data file 13, as explained for Additional data files 6 and 7, is from comparing the control parent vs. parent experiment (100 μM AsIII for 2 h) to the *arr1Δ *profiling experiment treated in parallel. Additional data file 14 contains a table of genes ('Minus' list) that fail to be induced or repressed, compared to the parent experiment, in the *arr1Δ *experiment after treatment with 100 μM AsIII for 2 h. Additional data file 15 contains the self-organized clustering of specific deletion and parent strain experiments (*yap1Δ *vs. *yap1Δ *2 h 100 μM AsIII, *cad1Δ *vs. *cad1Δ *2 h 100 μM AsIII, *rpn4Δ *vs. *rpn4Δ *2 h 100 μM AsIII, *arr1Δ *vs. *arr1Δ *2 h 100 μM AsIII, parent vs. parent with 2 h 100 μM AsIII, as well as the parent strain vs. each deletion strain without arsenic). Additional data files 16, 17, 18 and 19 contain the gene lists of differential expression in knockout strains *yap1Δ*, *cad1Δ*, *rpn4Δ *and *arr1Δ*, respectively, compared to the parent without arsenic treatment. Additional data file 20 contains every gene mentioned in this paper and the corresponding gene product descriptions. The primary microarray data will be submitted to the Gene Expression Omnibus (GEO) database at [80].

## Supplementary Material

Additional data file 1 The dose-response curve of *S. cerevisiae *strain, BY4741 Click here for additional data file

Additional data file 2 A self-organized tree of arsenite treated yeast experiments and a table depicting the numbers of significant genes Click here for additional data file

Additional data file 3 The primary raw cDNA data from all the experiments Click here for additional data file

Additional data file 4 The primary raw data for all the deletion experiments Click here for additional data file

Additional data file 5 The ranked arsenite sensitivity (phenotypic profiling) data Click here for additional data file

Additional data file 6 Genes two-fold or more differentially expressed after arsenite in the Yap1 deletion strain compared to the parent Click here for additional data file

Additional data file 7 Genes two-fold or more differentially expressed after arsenite in the Cad1 deletion strain compared to the parent Click here for additional data file

Additional data file 8 Genes failing to be induced or repressed by arsenite in the Yap1 deleted strain Click here for additional data file

Additional data file 9 Genes failing to be induced or repressed by arsenite in the Cad1 deleted strain Click here for additional data file

Additional data file 10 Under arsenite-treated conditions, Yap1 might regulate Arr2 and Arr3, and does not regulate Rpn4 Click here for additional data file

Additional data file 11 Genes two-fold or more differentially expressed after arsenite in the Rpn4 deletion strain compared to the parent Click here for additional data file

Additional data file 12 Genes failing to be induced or repressed by arsenite in the Rpn4 deleted strain Click here for additional data file

Additional data file 13 Genes two-fold or more differentially expressed after arsenite in the Arr1 deletion strain compared to the parent Click here for additional data file

Additional data file 14 Genes failing to be induced or repressed by arsenite in the Arr1 deleted strain Click here for additional data file

Additional data file 15 Self-organized clustering of deletion strains with AsIII treatment and parent strain vs. deletion strains without arsenic Click here for additional data file

Additional data file 16 Gene list of two-fold differential expression in *yap1Δ* vs. parent without arsenic treatment Click here for additional data file

Additional data file 17 Gene list of two-fold differential expression in *cad1Δ* vs. parent without arsenic treatment Click here for additional data file

Additional data file 18 Gene list of two-fold differential expression in *rpn4Δ* vs. parent without arsenic treatment Click here for additional data file

Additional data file 19 Gene list of two-fold differential expression in *arr1Δ* vs. parent without arsenic treatment Click here for additional data file

Additional data file 20 A file of all the genes mentioned in the paper Click here for additional data file

## Figures and Tables

**Table 1:** Pathways enriched for genes significantly expressed in response to arsenic

Category	Differentially expressed genes	Pathway size	*p*-value	Significant
**KEGG pathway**				
Cell cycle reference pathway	8	87	0.9072	False
Galcatose metabolism	5	15	0.0391	False
**Glutathione metabolism**	**6**	**11**	**0.0014**	**True**
MAPK signaling pathway	7	55	0.609	False
**Methionine metabolism**	**8**	**11**	**1.07E-05**	**True**
Proteasome	9	30	0.0127	False
Purine metabolism	14	139	0.8991	False
Pyrmidine metabolism	8	80	0.8515	False
**Sulfur metabolism**	**7**	**7**	**7.15E-07**	**True**
Serine, threonine and glycine metabolism	8	25	0.0125	False
Citrate cycle	4	22	0.3345	False
Starch and sucrose	9	31	0.0159	False
Pyruvate	4	25	0.4292	False
Reductive carboxylate	5	16	0.0508	False
Second messenger signaling	3	19	0.472	False
Valine, leucine, isoleucine	2	13	0.5313	False
Circadian rhythm	2	19	0.7398	False
Porphyrin and chlorophyll metabolism	7	74	0.8782	False
**Selenoamino-acid metabolism**	**10**	**12**	**8.36E-08**	**True**
Ubiquitin-mediated proteolysis	2	29	0.9133	False
Cysteine metabolism	2	4	0.088	False
Fructose and mannose	6	15	0.0093	False
Carbon fixation	3	15	0.3207	False
Alanine and aspartate	2	24	0.8477	False
Glutamate	3	19	0.472	False
Methane	2	4	0.088	False
				
**Gene Ontology (biological process)**				
Biological process	72	436	0.0244	False
**Cell communication**	**72**	**270**	**<1.00E-008**	**True**
Cell growth and maintenance	47	268	0.0231	False
Cell surface linked signal transduction	14	91	0.3197	False
Developmental processes	5	32	0.4233	False
**Heat-shock response**	**14**	**22**	**5.40E-08**	**True**
Intracellular signaling	9	47	0.1635	False
Serine threonine kinase signaling	5	38	0.5815	False
Signal transduction	26	172	0.2656	False
ATPase	3	78	0.9988	False
Cyclin	4	29	0.5499	False

Transcript profiling reveals that arsenic affects glutathione, methionine, sulfur, selenoamino-acid metabolism, cell communication and heat-shock response. Genes were categorized by KEGG pathway and Simplified Gene Ontology. In total, 829 genes out of 6,240 had a significant alteration in expression in at least one experimental condition. Along with the size of each functional category, a statistical measure for the significance of the enrichment was calculated by using a hypergeometric test. The level of significance for this test (True-shown in bold, False) was determined using the Bonferroni correction, where the α value is set at 0.05 and 27 and 11 tests were done for KEGG pathway and Simplified Gene Ontology, respectively.

**Table 2:** Genes affiliated to sulfur metabolism

Gene	Open reading frame	AsIII	Cd^2+^	H_2_O_2_	Enzyme
			
		Fold change			
Sulfate assimilation					
*MET3*	YJR010W	5.0-20.0	10.8	*-1.0-3.0*	ATP sulfurylase
*MET14*	YKL001C	2.0-14.0	9.6	*-2.0-3.0*	APS kinase
*MET16*	YPR167C	2.0-12.0	9.1	NC	PAPS reductase
*MET22 (*0.5 h, 1 mM AsIII)	YOL064C	3.3	2.5	NC	Diphosphonucleoside phosphohydrolase
*MET10*	YFR030W	3.0-5.0	5.2	NC	Sulfite reductase alpha
*MET5/ECM17*	YJR137C	2.0-5.0	4.5	*-2.0-4.0*	Sulfite reductase beta
*MET1/20*	YKR069W	NC	6	NC	Uroporphyrinogen III methylase
*MET8*	YBR213W	NC	7	*-2.0-4.0*	Siroheme synthase
					
Sulfide incorporation and transulfuration pathways					
*MET2*	YNL277W	4.0-3.0	4.6	NC	Homoserine transacetylase
*MET25/17*	YLR303W	NC	4.8	NC	O-Acetylhomoserine sulfhydrylase
*STR4/CYS4*	YGR155W	2.0-2.7	2.5	*-2.0-3.0*	Cystathionine B-synthase
*STR1/CYS3 *(4 h, 100 μM AsIII)	YAL012W	*-3.5*	13.4	NC	Cystathionine-lyase
*STR3*	YGL184C	2.2-3.9	13.5	2.0-4.0	Cystathionine G-synthase
*YFR055W*	YFR055W	*-9.0-5.0*	*-1.1*	NC	Cystathionine-lyase
					
Methionine and AdoMet biosynthesis					
*MET6*	YER091C	1.0-3.5	NC	*-2.0-5.0*	N5-Methyltetrahydrofolate homocysteine transferase
*MET7*	YOR241W	NC	*-1.6*	NC	Tetrahydrofolyl polyglutamate synthase
*MET13*	YGL125W	NC	NC	*-1.0-3.0*	Methylene tetrahydrofolate reductase
*SAM1 *(4 h, 100 μM AsIII)	YLR180W	2.5	NC	*-2.0-9.0*	AdoMet synthetase
*SAM2 *(4 h, 100 μM AsIII)	YDR502C	3.8	NC	*-2.0-4.0*	AdoMet synthetase
*MHT1*	YLL062C	5.0-2.8	10.6	NC	S-methylmethionine: homocysteine S-methyltransferase
					
Sulfur compound uptake					
*SUL1*	YBR294W	5.4-2.4	20	NC	Sulfate transporter
*SUL2*	YLR092W	2.5-2.8	3	NC	Sulfate transporter
*MUP1 *(2 h and 4 h, 100 μM AsIII)	YGR055W	5.0-14.0	2	*-1.0-4.0*	Methionine permease, high affinity
*MUP3*	YHL036W	8.0-7.0	7		Methionine permease, low affinity
					
Regulatory genes					
*MET4 *(2 h, 100 μM AsIII)	YNL103W	2	1.5	NC	bZIP
*MET28*	YIR017C	NC	5	NC	bZIP
*CBF1*	YJR060W	NC	*-2*	NC	bHLH
*MET30*	YIL046W	5.0-1.6	7	NC	WD40 repeats F box
*MET31*	YPL039W	NC	1	*-1.0-7.0*	Zinc finger
*MET32*	YDR253C	6.0-3.6	14	*-1.0-4.0*	Zinc finger

Arsenic treatment stimulates a sulfur response in yeast. Gene expression data comparisons between arsenic, cadmium, and H_2_O_2_-treated *Saccharomyces cerevisiae *reveal arsenic and cadmium mediated sulfur responses, but none with hydrogen peroxide. AsIII column, 2 h, 100 μM and 0.5 h, 1 mM (combined biological replicates), unless noted; cadmium column, 1 h, 1 mM [63]; H_2_O_2 _column, 10, 20, 40, 60, 120 min, 400 μM [62]. Numbers in ordinary typeface denote induction; (-) and italicized numbers denote repression; NC, no change.
