# Supplementary figures and images for: Integrating phenotypic and expression profiles to map arsenic-response networks
Source: Genome Biol. 2004 Nov 29;5(12):R95. doi: 10.1186/gb-2004-5-12-r95 (PMC545798; doi:10.1186/gb-2004-5-12-r95)

A.

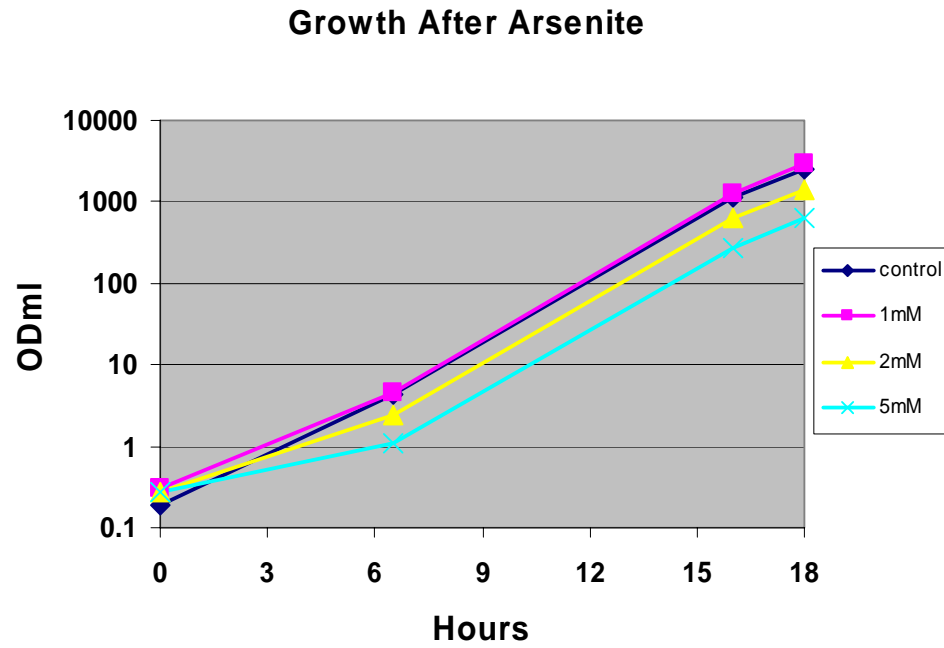

**Survival to Arsenite**

B.

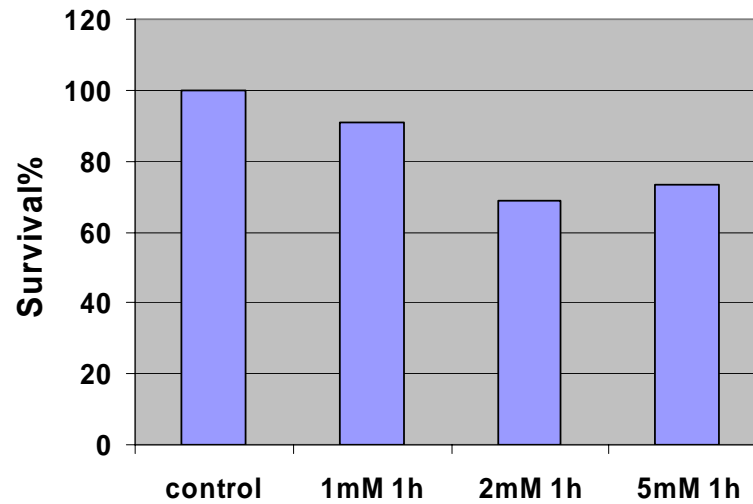

Additional data file 1. The dose response curve of *S. cerevisiae* strain, BY4741.

Supplement: Additional data file 1 — The dose-response curve of S. cerevisiae strain, BY4741 [file gb-2004-5-12-r95-s1.pdf]
