# Supplementary material for: Integrating phenotypic and expression profiles to map arsenic-response networks
Source: Genome Biol. 2004 Nov 29;5(12):R95. doi: 10.1186/gb-2004-5-12-r95 (PMC545798; doi:10.1186/gb-2004-5-12-r95)
Supplement: Additional data file 2 — A self-organized tree of arsenite treated yeast experiments and a table depicting the numbers of significant genes [file gb-2004-5-12-r95-s2.pdf]

Additional data file 2. Self-organized tree of arsenite treated yeast experiments and table depicting the numbers of significant genes.

A

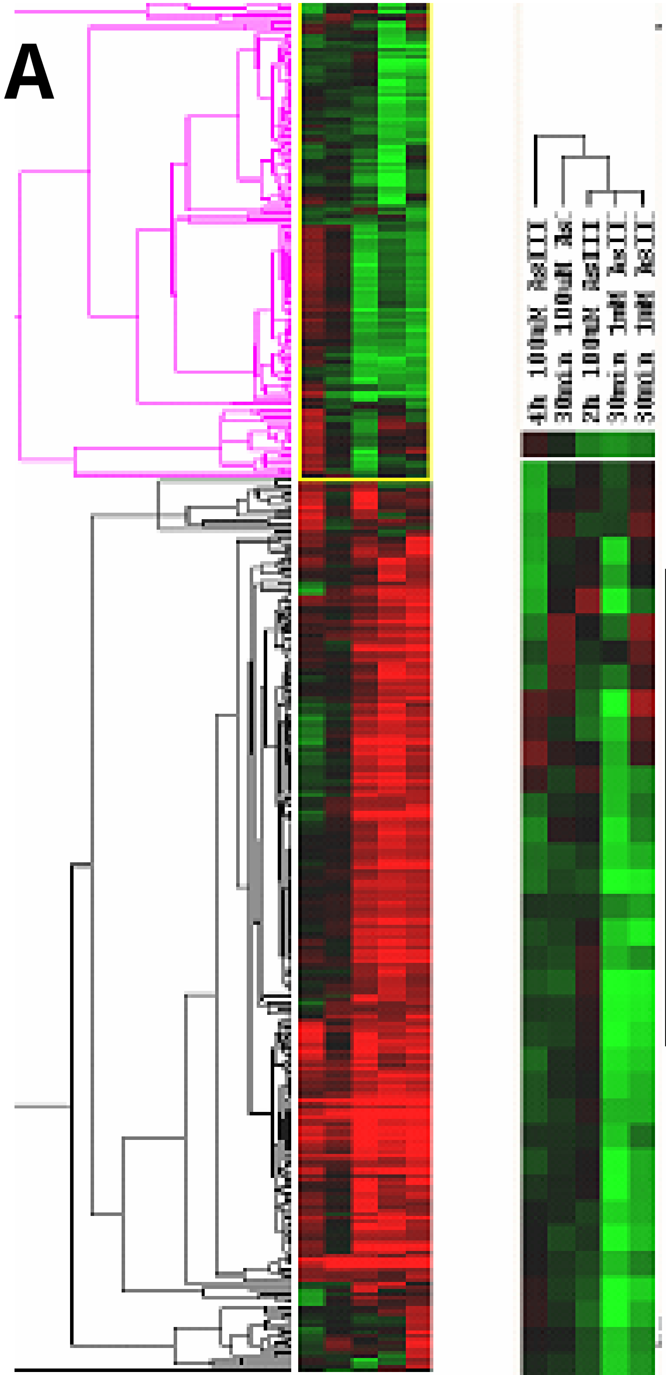

B

Differentially Expressed Genes: 3 out of 4 hybridizations

Experiments	95% Confidence Interval			99% Confidence Interval		
	UP	DOWN	Total	UP	DOWN	Total
30 min. 1mM AsIII replicate A	289	209	498 (8.3%)	150	58	208(3.3%)
30 min. 1mM AsIII replicate B	386	232	618 (10.3%)	211	66	277 (4.5%)
30 min. 100uM AsIII	68	44	112 (1.9%)	48	17	65 (1.04%)
2 hours 100uM AsIII	262	170	432 (7.2%)	84	33	117 (1.9%)
4 hours 100uM AsIII	190	75	265 (4.4%)	81	13	94 (1.5%)
