# Supplementary material for: Integrating phenotypic and expression profiles to map arsenic-response networks
Source: Genome Biol. 2004 Nov 29;5(12):R95. doi: 10.1186/gb-2004-5-12-r95 (PMC545798; doi:10.1186/gb-2004-5-12-r95)
Supplement: Additional data file 10 — Under arsenite-treated conditions, Yap1 might regulate Arr2 and Arr3, and does not regulate Rpn4 [file gb-2004-5-12-r95-s10.pdf]

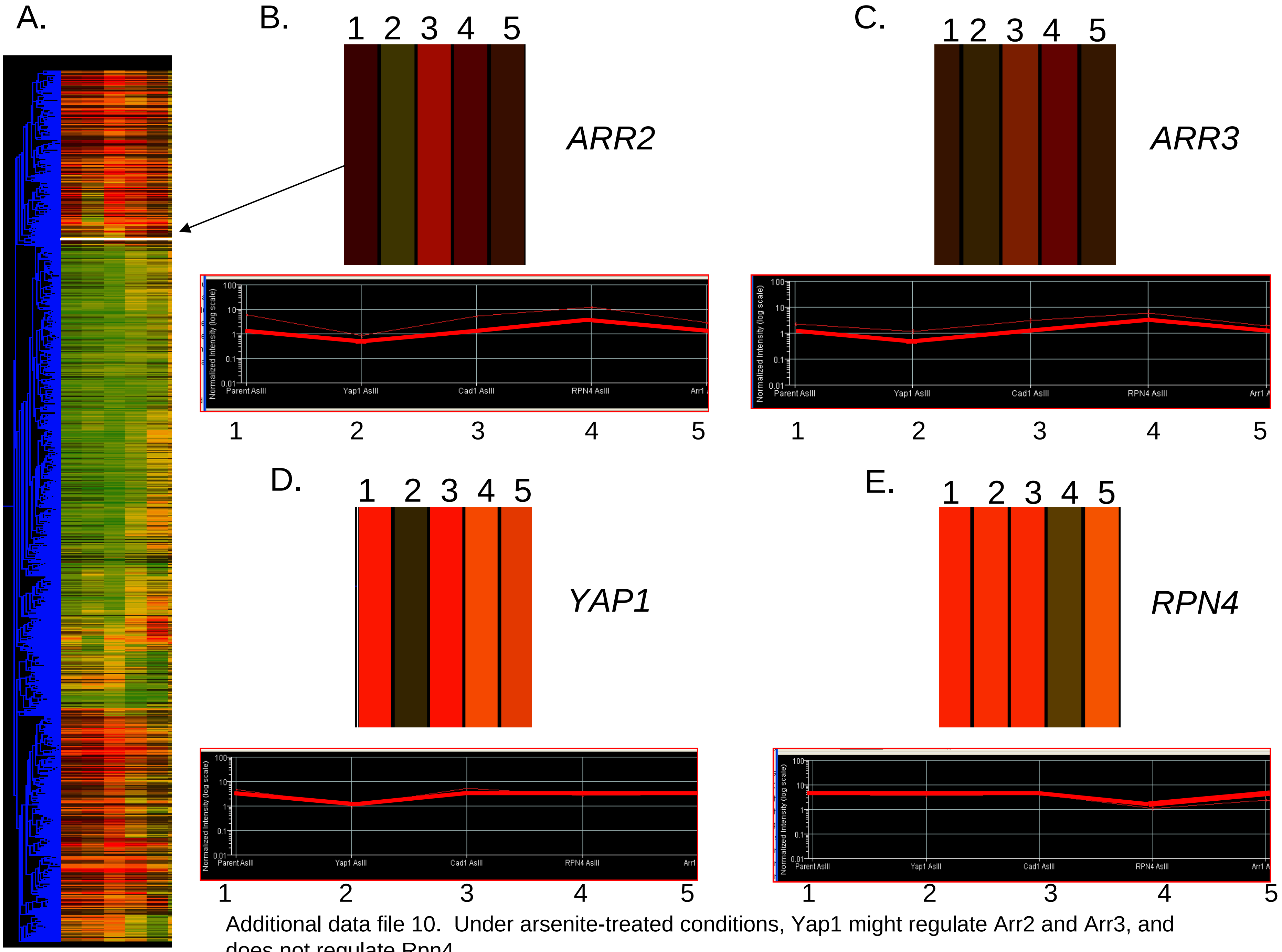

Additional data file 10. Under arsenite-treated conditions, Yap1 might regulate Arr2 and Arr3, and does not regulate Rpn4.
