# Supplementary material for: Integrating phenotypic and expression profiles to map arsenic-response networks
Source: Genome Biol. 2004 Nov 29;5(12):R95. doi: 10.1186/gb-2004-5-12-r95 (PMC545798; doi:10.1186/gb-2004-5-12-r95)

Additional data file 15. Self-organized clustering of deletion strains with AsIII treatment and parent strain vs. deletion strains without arsenic.

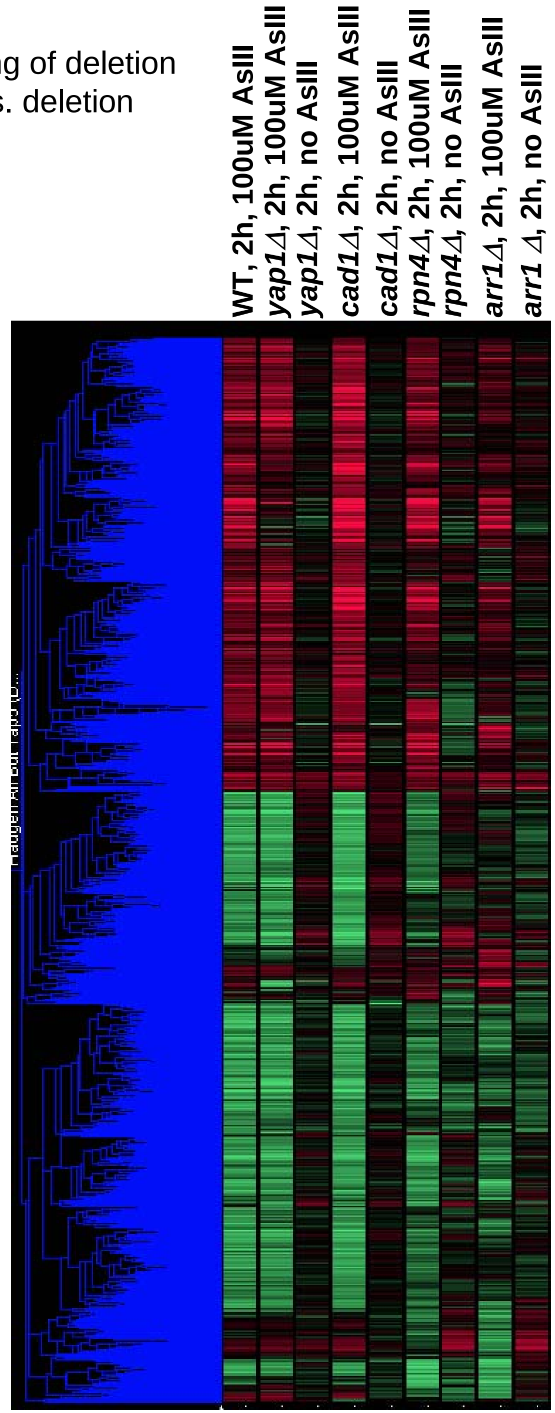

Supplement: Additional data file 15 — Self-organized clustering of deletion strains with AsIII treatment and parent strain vs. deletion strains without arsenic [file gb-2004-5-12-r95-s15.pdf]
